# Post-kyphoplasty myelopathy, an unusual presentation of post-operative anterior spinal cord infarct: A case report

**DOI:** 10.1016/j.inpm.2023.100267

**Published:** 2023-07-04

**Authors:** Shuo Li, Jenessa Lemon, Mohamed Ibrahim, Kaitlyn Mi, Sierra Ferguson, Kermit Rust, James Homan

**Affiliations:** a Department of Radiology, University of Kansas School of Medicine, Wichita, KS, USA; b College of Osteopathic Medicine, Kansas City University, Kansas City, MO, USA; c The Dartmouth Institute of Health Policy and Clinical Practice, Lebanon, NH, USA

**Keywords:** anterior spinal artery, Surfer myelopathy, Kyphoplasty, Bone cement

## Abstract

**Highlights:**

To discuss a rare complication of prone positioning during kyphoplasty.

To compare two rare causes of anterior spinal artery infarct secondary to prone positioning: Surfer Myelopathy and post-kyphoplasty myelopathy.

**Background:**

Kyphoplasty is a common, minimally invasive procedure performed to restore vertebral body structure and relieve pain in insufficiency fractures that are refractory to conservative treatments. Complications are infrequent, but typically arise from epidural hematoma, cement embolism, or cement extravasation causing stenosis within the spinal canal or neural foramina. In this case, we discuss a rare complication involving a spinal cord infarct developing several levels above the level of intervention due to compression of the anterior spinal artery.

**Case presentation:**

A 71-year-old female with kyphotic deformity and midthoracic compression fractures underwent a procedurally uneventful T12 kyphoplasty. Pre-procedure MRI demonstrated T12 superior endplate compression deformity with mild retropulsion of the superior endplate. Chronic T6 and T8 compression fractures with kyphotic deformity were also seen. Shortly after the procedure, she developed right leg pain and numbness progressing to profound weakness. She was taken immediately for CT scan of the thoracolumbar spine which was negative for cement extravasation, and subsequent MRI was negative for epidural hematoma. The MRI did show a peculiar finding of spinal cord infarct from T8 to the conus with punctate hemorrhage at T11.

**Conclusions:**

It is postulated that the incomplete cord infarct in this patient occurred due to compression of the anterior spinal artery or radicular arteries during positioning in the setting of kyphotic deformity and posterior osteophyte. The dysmorphic changes seen at T8 may have behaved similarly to a disc herniation in compressing the spinal artery in a prone position.

## 1 Introduction

Vertebral balloon augmentation with polymethylmethacrylate bone cement, or kyphoplasty, is a common minimally invasive procedure usually resorted to in the case of failure of conservative treatment of osteoporotic vertebral compression fractures [1]. The main objective of kyphoplasty is to decrease pain and reduce further vertebral body collapse [2,3]. Complications are uncommon, and most are not directly related to the procedure. If we exclude general procedural and anesthesia-related complications, most adverse outcomes linked to kyphoplasty are due to cement extravasation [4]. This is asymptomatic in most cases. However, cement leakage into the spinal canal or neuro-foramina could potentially cause neurological symptoms from local compression [5], pulmonary embolism from cement traveling through the valveless vertebral venous plexus to the lungs, or a stroke if a patent foramen ovale is present [3]. Embolization into the arterial system has also been reported, including emboli to the anterior spinal artery causing anterior spinal artery syndrome [2,3,6,7].

In this case, we discuss an even more rare complication of kyphoplasty, likely precipitated simply by prone positioning rather than any of the above factors. Studies describing anterior spinal artery ischemia due to prone positioning are limited. It is thought that prone positioning, especially with hyperextension of the spine, causes decreased spinal cord perfusion due to partial occlusion of the inferior vena cava and it's collaterals leading to increased spinal venous pressure [8,9]. Ischemia can be further exacerbated by radicular artery compression as it travels through an intervertebral foramen obstructed by osteophytes and kyphotic change, as seen in our patient.

We compare our findings to a similar, uncommon condition found in novice surfers. These patients present with “Surfer Myelopathy” after spending an atypically long time in the prone position floating on a board in the ocean [[10], [11], [12], [13]]. While attempting to stand, these surfers repeatedly hyperextend their spines, with potential further hyperextension upon contact with a wave. This activity is repeated without adequate time for reperfusion of the spine, resulting in ischemia in the distribution of the anterior spinal artery [9,13].

## 2 Case report

**Fig. 1:**
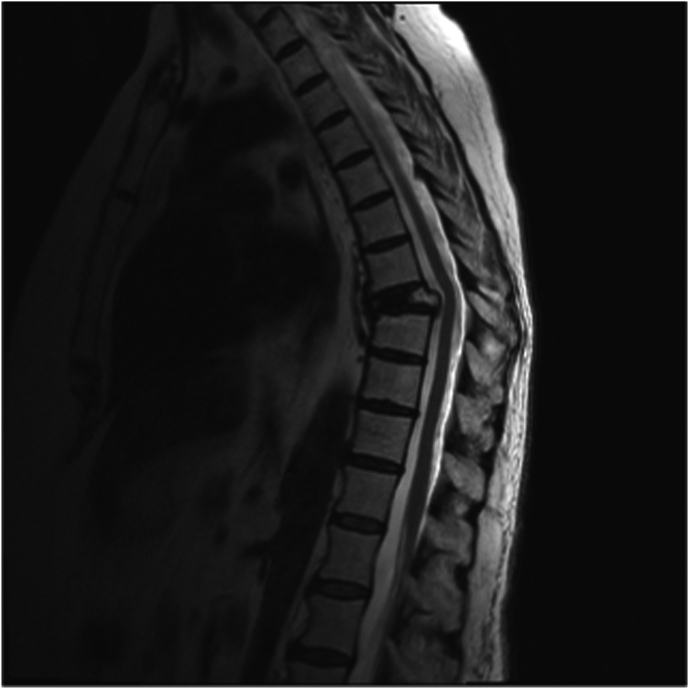
Sagittal non-fat saturated T2 weighted image MRI of the thoracic spine 5 months prior to intervention showing normal thoracic spinal cord without abnormal signal. No evidence of cord edema.

**Fig. 2:**
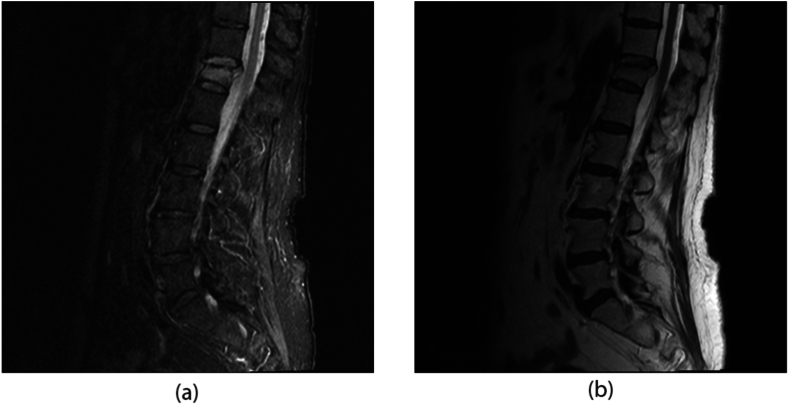
Sagittal STIR (2a) and non-fat saturated T2 (2b) weighted images 2 weeks prior to intervention showing an acute compression fracture at T12. The visualized portions of the lower thoracic spinal cord is normal in appearance.

**Fig. 3:**
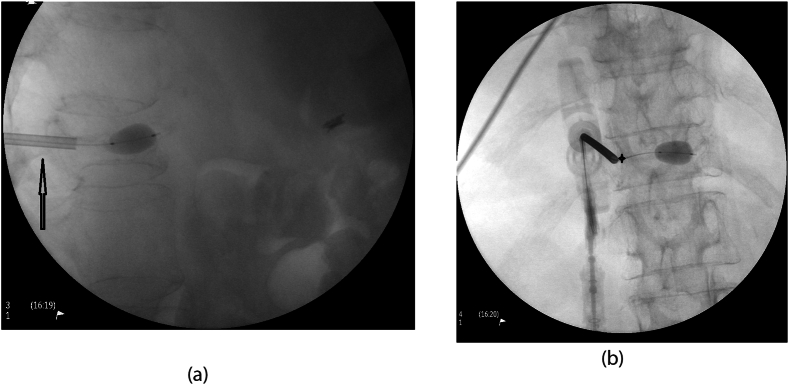
Sagittal intraoperative fluoroscopy (3a) demonstrating the trocar in the vertebral body with an inflated balloon. Note that the trocar remains between the margins of the pedicle. The inferior margin is indicated by the black hollow arrow. Posterior to anterior (PA) intraoperative fluoroscopy (3b) demonstrating transpedicular approach of the trocar. The cannula is lateral to the medial border of the pedicle, indicated by the black hollow star.

A 71-year-old female with past medical history of hypertension, hyperlipidemia, asthma, multiple myeloma, hypothyroidism, and gastroesophageal reflux disease presented with four weeks of severe mid back pain and midline tenderness to palpation at the thoracolumbar junction. Pre-procedure MRI demonstrated T12 superior endplate compression deformity with mild retropulsion of the superior endplate. Chronic T6 and T8 compression fractures with kyphotic deformity were also seen. The patient underwent a procedurally uneventful left unipedicular T12 kyphoplasty using a curved 13 gauge Stryker kyphoplasty kit. Three to five cubic centimeters of bone cement were used, and the duration of the procedure was 35 minutes. Blood pressure throughout the procedure was 145/55 at initiation of sedation and 125/50 at end time. During sedation, there was a dip in the blood pressure following an additional dose of Versed 1mg and Fentanyl 50mcg. The lowest blood pressure was observed at 95/45. Heart rate was noted to be sinus tachycardia at 105–130, oxygen saturations were well maintained on 2L O_2_ at 97%–99%. At the end of the procedure the patient began to complain of right leg pain and numbness; progressing to profound weakness. She experienced profound sensory deficits for 55 minutes following the procedure (see Fig. 1, Fig. 2, Fig. 3).

### 2.1 Investigations

**Fig. 4:**
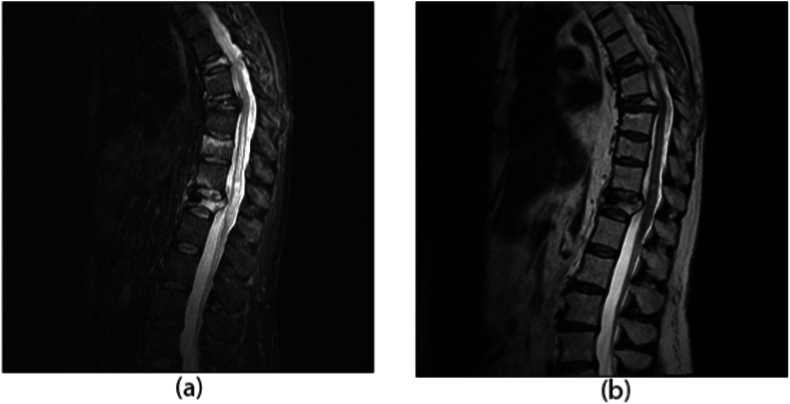
Sagittal STIR (4a) and non-fat saturated T2 (4b) weighted images within 1 h following T12 kyphoplasty. There is now increased signal intensity in the lower thoracic spinal cord which slightly expanded appearance on the T2WI. Additional acute compression fractures are also seen. Retropulsion of the posterior element of T12 is not changed from the pre-op images.

**Fig. 5:**
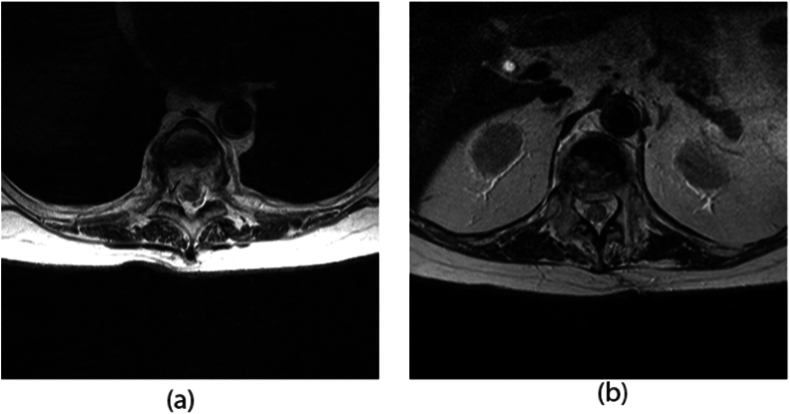
T2 weighted, non-fat saturated axial images were obtained at the level of intervention prior to (5a) and following (5b) kyphoplasty.

At the time of the onset of these neurologic symptoms, the differential consideration included spinal stenosis from cement extravasation versus epidural hematoma. The patient was taken immediately for CT of the thoracolumbar spine, which was negative for extravasation. Immediate follow up MRI of the region found no evidence of epidural hematoma. However, MRI did show a spinal cord infarct evidenced by a T2 hyperintense signal in the anterior spinal artery territory from T8 to the conus with punctate hemorrhage at T11 (see Fig. 4, Fig. 5).

### 2.2 Treatment

The patient was emergently transferred to a regional medical center where neurosurgery and neurology were consulted. Neurology started her on high-dose steroids, neurosurgery reviewed the films and recommend no further intervention. MRI was repeated and confirmed the cord infarct at T8 with hemorrhage, and the steroids were discontinued at that time. During the course of her admission, the patient had minimal improvement in her right lower extremity weakness. Her sensory exam improved somewhat, but she remained significantly diminished. She was transferred for inpatient rehabilitation and received a short course of steroid therapy.

### 2.3 Outcome and follow up

**Fig. 6:**
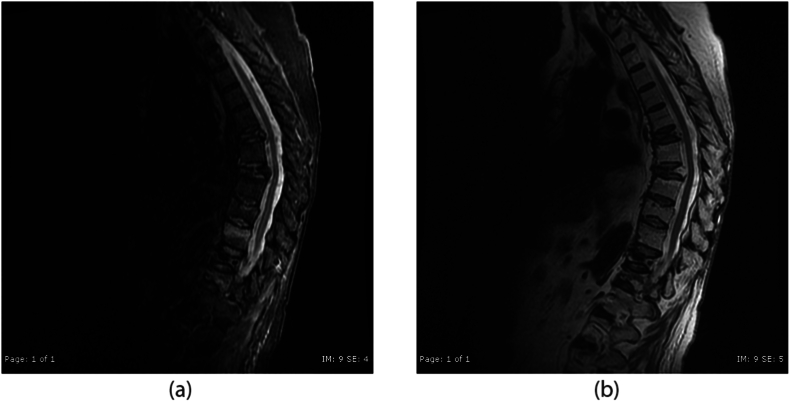
Sagittal STIR (6a) and non-fat saturated T2 (6b) weighted images 1 month after T12 kyphoplasty. There is slight residual hyperintensity of the caudal thoracic spinal cord, which is improved from the previous figure.

Three months later, the patient was then seen in the office. She had significant improvement in right lower extremity strength. She walked with a cane and an ankle-foot brace for foot drop. Otherwise, she lives a fully functional life, and is independent with activities of daily living (see Fig. 6).

## 3 Discussion

The MRI findings in this case reveal a spinal cord infarct that developed several levels above the level of intervention due to compression of the anterior spinal artery. The data surrounding anterior spinal artery ischemia from either hypoperfusion or occlusion is limited. It is postulated that the incomplete cord infarct in this patient occurred due to compression of the anterior spinal artery or radicular arteries during positioning in the setting of kyphotic deformity and posterior osteophyte. Literature suggests the cause of anterior spinal artery ischemia due to degeneration of the spine or disc herniation is a rare event [14]. The retropulsed and kyphotic changes seen at T8 may have behaved similarly to a disc herniation in compressing the spinal artery in a prone position.

In most of the reported cases of anterior spinal artery syndrome, patients developed paraplegia and loss of pain/temperature below the level of the affected segment [6,7]. Our patient developed right sided pain and numbness shortly after an uneventful kyphoplasty. She also experienced delayed right sided weakness that later resolved. This is possibly the 2nd reported case of a reversible anterior spinal cord syndrome, after the case reported by Bredow J et al., in 2014 [7]. Similar to the Bredow J et al. case, no evidence of cement leakage was present on follow up imaging.

The exact mechanism of this reversible anterior spinal cord infarct remains unknown. Several etiologies could potentially cause anterior spinal cord syndrome. These may include vasculitides, microangiopathies, fistulas or arterial embolization of the anterior spinal artery [7]. Another possibility is intraoperative injury to or compression of the radiculomedullary artery or Artery of Adamkiewicz. We do not suspect injury to these vessels because of the transpedicular approach maintained throughout the duration of the procedure.

We hypothesize in our case that prone positioning could potentially be the main culprit. The presentation is comparable to a rare neurologic condition found in novice surfers. Patients with Surfer's myelopathy are usually beginner surfers who do not typically assume prolonged prone positioning. They consequently develop anterior spinal cord syndrome after a long day learning to surf, floating prone on a surfboard [10]. Similarly, our patient who was not accustomed to prone positioning due to limitations from chronic back pain was placed in this position for an extended period of time for the procedure, resulting in atypical pressure on anterior spinal artery.

While anterior spinal artery ischemia is an emergent and serious complication, it is felt to be an extremely rare event, and no pre-kyphoplasty screening is necessary in the future. However, effort should be made to prevent general vascular complications and neuropathies associated with prolonged prone positioning. Padding bony prominences, strategic positioning of the limbs, and avoiding intraoperative hypotension can minimize risks associated with surgical prone positioning [15]. It is reasonable to assume that applying similar precautions, in addition to minimizing time in a prone, hyperextended position, may minimize risk for complications in patients undergoing kyphoplasty with structural abnormalities of the spine.

## Funding

This research did not receive any specific grant from funding agencies in the public, commercial, or not-for-profit sectors.

## Declaration of competing interest

The authors declare that they have no known competing financial interests or personal relationships that could have appeared to influence the work reported in this paper.
